# Therapeutic Candidates for Alzheimer’s Disease: Saponins

**DOI:** 10.3390/ijms241310505

**Published:** 2023-06-22

**Authors:** Ruifeng Zhang, Miao Zeng, Xiaolu Zhang, Yujia Zheng, Nuan Lv, Luming Wang, Jiali Gan, Yawen Li, Xijuan Jiang, Lin Yang

**Affiliations:** School of Integrative Medicine, Tianjin University of Traditional Chinese Medicine, Tianjin 301617, China; zrf18235275625@163.com (R.Z.); hbzyzm39@163.com (M.Z.); xiaoluzhang0@foxmail.com (X.Z.); 15776605761@163.com (Y.Z.); m15531492601@163.com (N.L.); m18937271730@163.com (L.W.); 17320291675@163.com (J.G.); lyw18711213053@163.com (Y.L.)

**Keywords:** Alzheimer’s disease, saponin, amyloid beta, inflammation, oxidative stress, apoptosis

## Abstract

Drug development for Alzheimer’s disease, the leading cause of dementia, has been a long-standing challenge. Saponins, which are steroid or triterpenoid glycosides with various pharmacological activities, have displayed therapeutic potential in treating Alzheimer’s disease. In a comprehensive review of the literature from May 2007 to May 2023, we identified 63 references involving 40 different types of saponins that have been studied for their effects on Alzheimer’s disease. These studies suggest that saponins have the potential to ameliorate Alzheimer’s disease by reducing amyloid beta peptide deposition, inhibiting tau phosphorylation, modulating oxidative stress, reducing inflammation, and antiapoptosis. Most intriguingly, ginsenoside Rg1 and pseudoginsenoside-F11 possess these important pharmacological properties and show the best promise for the treatment of Alzheimer’s disease. This review provides a summary and classification of common saponins that have been studied for their therapeutic potential in Alzheimer’s disease, showcasing their underlying mechanisms. This highlights the promising potential of saponins for the treatment of Alzheimer’s disease.

## 1. Introduction

Alzheimer’s disease (AD), the leading cause of dementia, is a progressive neurodegenerative disease [1] that is clinically characterized by memory loss, cognitive impairment, and behavioral disturbances [2]. Since its discovery in 1906, AD has emerged as one of the most costly, fatal, and burdensome diseases of this century [3]. The pathogenesis of AD involves various biological processes [4] involving the abnormal deposition of amyloid beta peptide (Aβ) [5], the accumulation of neurofibrillary tangles (NFTs) [6], neuroinflammation [7], neuronal apoptosis [8], neurotransmitter abnormities [9], and oxidative stress [10]. Despite considerable efforts, drug discovery for the treatment of AD has been slow, with only acetylcholinesterase (AChE)/butyrylcholinesterase (BChE) inhibitors [11] such as galantamine, donepezil, tacrine, and rivastigmine currently available as therapies [12]. However, these treatments only delay the onset of symptoms and cannot halt disease progression and are often associated with significant side effects [13]. Therefore, the development of new therapeutic drugs is urgently needed. Saponins, a type of natural compound, have been extensively studied for their various pharmacological properties [14]. Of particular interest is their potential to enhance learning and memory in individuals with AD [15].

**Figure 1:**
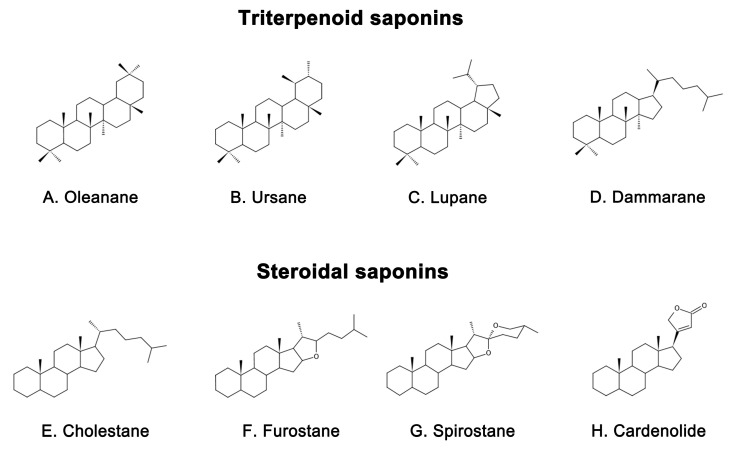
Representative saponin skeleton structure of triterpenoid saponins include (**A**) Oleanane, (**B**) Ursane, (**C**) Lupane, (**D**) Dammarane and steroidal saponins include (**E**) Cholestane, (**F**) Furostane, (**G**) Spirostane, (**H**) Cardenolide.

Saponins are naturally occurring compounds that are widely distributed in various plants [16], and they can be divided into two major groups based on their chemical structure: triterpenoid saponins and steroidal saponins [17]. Triterpenoid saponins are further subdivided into tetracyclic triterpenes and pentacyclic triterpenes and are mainly found in plants such as Pentaaceae, Leguminosae, Poria, and Platycodonaceae [18]. The main saponin skeletons of the triterpenoid saponins include dammarane, oleanane, ursane, and lupane (Figure 1A–D) [19]. Steroidal saponins, on the other hand, are mainly found in plants such as Dioscoreaceae, Liliaceae, and Scrophulariaceae [20]. The main saponin metaskeletons of steroidal saponins include spirostane, furostane, cholestane, and cardenolide (Figure 1E–H) [21]. Saponins possess multiple bioactivities, such as reduction of amyloid beta (Aβ) deposition [22], inhibition of tau protein phosphorylation [23], antioxidation [24], antiapoptosis [25], and anti-inflammation [26]. These properties make saponins promising therapeutic candidates for AD and other neurological disorders [27]. Meanwhile, the diversity of saponins found in different plants [28] makes them a valuable source of potential drugs for the treatment of AD. However, there is a current lack of comprehensive and systematic review in this field. To address this gap, we thoroughly searched the relevant literature in the major databases, including PubMed and Web of Science, using keywords “Alzheimer’s disease” or “AD” and “saponin”. By browsing all the relevant studies from May 2007 to May 2023, excluding review articles, 63 references were selected, involving 40 saponins extracted from different herbs. The selected references are known to be effective in the treatment of AD, present clear mechanisms, authentic and reliable information, and contain the latest findings in the field. The studies highlighted that saponins have exhibited various beneficial effects such as reducing Aβ levels, reducing NETs, exerting antioxidative, antiapoptotic, anti-inflammatory, and increased neurotransmitter-enhancing effects [25]. The underlying mechanisms behind these effects include reducing amyloid precursor protein (APP) production [29], improving tau protein phosphorylation [30], and reducing reactive oxygen species (ROS) generation [31], inhibiting the apoptotic and inflammatory signaling pathways [31,32], and increasing neurotransmitter expression [33]. This comprehensive review sheds light on the potential saponins as therapeutic agents for AD and related neurological disorders.

## 2. Mechanism of Saponins in Treating Alzheimer’s Disease

### 2.1. Inhibition of Aβ Deposition and Neurotoxicity

Aβ is a peptide that is a major component of the senile plaques found in the brains of people with AD [34]. Its accumulation is highly neurotoxic and is considered a hallmark of AD [35], which can result in impaired cognitive function, including spatial memory [36]. Aβ is generated from the APP and is typically enclosed by microglia and dystrophic synapses that aggregate around neurons [37,38]. APP is a transmembrane protein located in the synapse of neurons, which can be cleaved by both amyloid and nonamyloid pathways [39]. In the nonamyloid pathway, APP is sequentially cleaved by α-secretase (mainly ADAM10) and γ-secretase [40], resulting in P3 peptide (P3), C83 carboxy-terminal fragment (C83), APP intracellular domain (AICD), and soluble amyloid precursor protein-α (sAPPα) with beneficial neurotrophic effects [41]. Conversely, in the amyloid pathway, APP is first cleaved by β-secretase and sAPPβ is secreted [42]. Subsequently, γ-secretase cleaves the C-terminal fragment (C99) of the residual APP and eventually leads to the release of peptides of different lengths [43]. The most prevalent of them are Aβ1-40 and Aβ1-42 [44], which are neurotoxic fragments capable of oligomerization, aggregation, and subsequent plaque formation [45]. In vivo, Aβ is degraded by a variety of proteases, most notably insulin-degrading enzymes (IDE) and neprilysin (NEP) [46]. However, the production or activity of these clearance enzymes may decrease with age, leading to a failure to clear Aβ in a timely manner [47]. This phenomenon has been linked to the reduced activity of the peroxisome proliferator-activated receptor γ (PPARγ) [48], which is a transcription factor that regulates the expression of IDE and Bace1, reduces Aβ production [49], promotes Aβ clearance [50], and exerts neuroprotective effects [51]. An imbalance between Aβ production and clearance is responsible for the accumulation of Aβ in the brain [52]. In order to develop novel treatments for AD, it may be beneficial to inhibit Aβ production, enhance its clearance, or directly combat its neurotoxicity [53].

Natural saponins have shown potential in affecting Aβ metabolism through different pathways [54]. For example, some natural saponins are able to inhibit the formation of Aβ by reducing APP production. Ginsenoside Rg1, a tetracyclic triterpenoid saponin extracted from ginseng [55], has been shown to reduce Aβ deposition in APP/presenilin 1(PS1) double transgenic AD model mice by lowering APP levels [56]. Pharmacokinetic studies have also shown that ginsenoside Rg1 can cross the blood–brain barrier (BBB) and the blood–cerebrospinal fluid barrier (BCSFB), which provides theoretical support for its ability to improve learning and memory abilities [57]. Xanthoceraside, another tetracyclic triterpenoid saponin, extracted from the bark of the Xanthoceras sorbifolia Bunge commonly used in traditional Chinese medicine (TCM) to treat rheumatism [58]_,_ has been shown to reduce APP protein levels and Aβ deposition in the cerebral cortex and hippocampus, thereby ameliorating cognitive function dysfunction in an AD mouse model induced by Aβ intracerebroventricular (ICV) injection [59]. In addition, ginsenoside Rh2, another ginseng derivative used in TCM, has been reported to regulate APP expression by reducing cholesterol and lipid raft levels. This ultimately led to an increase in sAPPα levels and a decrease in Aβ concentrations [60].

Most saponins have been shown to inhibit Aβ deposition by modulating the activity of APP-processing enzymes, such as ginsenoside Rg1 [61], RAPO-1-3 [62], onjisaponin B [62], pseudoginsenoside-F11 (PF11) [63], theasaponin E1 [64], anginsenoside (20S)-Rg3 [65], and ginsenoside C-K (CK) [66]. Ginsenoside Rg1 reduces the γ-secretase responsible for Aβ production by attenuating the Aβ-mediated inhibition of cAMP response element-binding protein (CREB) phosphorylation and protein kinase A (PKA) activity [61]. It also upregulates ADAM10 while reducing BACE1 in Wistar rat models of AD induced by ovariectomy (OVX) and D-galactose (D-gal) [67]. In sAPPα-transfected HT22 cells and neuroblastoma (SH-SY5Y) cells, ginsenoside Rg1 has been found to increase the levels of sAPPα and estrogen receptor (ER) α, elevate α-secretase activity, and decrease extracellular release of Aβ. Further studies have shown that these effects are mediated by the upregulation of phosphorylated extracellular signal-regulated kinase 1/2 (pERK1/2) and phosphorylated protein kinase B (pAkt) [68] but can be reversed by ER antagonists and potentially attenuated by inhibitors of protein kinase C (PKC), MAPK, and phosphatidyl 3-kinase (PI3K) [68]. Yuan Zhi (RAPO) is a TCM formulation that is commonly used to treat dementia due to its neuroprotective effects [69]. Its active ingredient, RAPO-1-3, and onjisaponin B, an acyl saponin with a similar constituent to RAPO-1-3, have been found to reduce Aβ production by promoting the degradation of APP protein through interference with the PS1/BACE1 interaction [62]. Another compound, PF11, which is a pentacyclic triterpene abundant in ginseng [70], has also demonstrated efficacy in inhibiting APP amyloidogenic processing. By reducing the expression of BACE1, PF11 reduced Aβ deposition and ameliorated cognitive impairment and synaptic dysfunction in SAMP8 mice [63]. Theasaponin E1, a pentacyclic triterpene extracted from green tea seeds [71], has been shown to reduce the production of Aβ by increasing the activity of NEP and ADAM10 in APP (SweAPP N2a) cells while inhibiting the expression of BACE1, APP, and PS1 [64]. Furthermore, CK, which is produced through the degradation of protopanaxadiol saponins by the gut microbiota [72], has been studied for its neuroprotective properties. Currently, it is primarily obtained by glycosyl hydrolysis of proto-ginsenoside diol-type saponins [73]. In scopolamine-induced ICR mice, CK was found to reduce the expression of BACE1 and PS1, increase IDE activity, reduce Aβ expression, and improve memory function [66]. In addition, ginsenoside (20S)-Rg3, a component of heat-processed ginseng, has been found to reduce Aβ levels by increasing phosphatidylinositol 4-kinase IIα(PI4KIIα) activity and ultimately decrease the expression of γ-secretase by decreasing the association of PS1 fragments and lipid rafts in cultured primary neurons and in the brains of an AD mouse model [65].

Aside from inhibiting Aβ production, certain saponins have been found to promote the clearance of Aβ. For example, minor ginsenoside F1, a trace ginsenoside derived from Panax ginseng [74], has been shown to effectively reduce Aβ plaques and alleviate cognitive impairment in APP/PS1 mice by enhancing the expression of pCREB [75]. Similarly, Bacopaside I (BS-I), a tetracyclic triterpene and standardized extract of Bacopa monnieri [76], has been shown to be neuroprotective and improve cognitive function [77]. The aglycones of Bacopa monnieri and its derivatives have good intestinal absorption and BBB permeability [78]. Recent studies suggest that BS-I induces sufficient innate immune stimulation and phagocytosis to promote amyloid clearance, thereby reducing amyloid plaque burden in APP/PS1 mice [79]. In addition, Aβ deposition in the brain is linked to the dysfunction of the endosomal–lysosomal system dysfunction [80], which is regulated by the transcription factor EB (TFEB) [81]. PF11 has been observed to increase Aβ clearance by promoting the mammalian target of rapamycin (mTOR)-dependent TFEB-mediated lysosome biogenesis and alleviating endosomal–lysosomal system dysfunction through the conversion of Rab5 to Rab7 [82].

It is interesting to note that some natural saponins do not directly target key enzymes involved in Aβ metabolism, but instead act as natural agonists of peroxisome proliferator-activated receptor gamma (PPARγ) [83]. For example, in N2a/APP695 cells, ginsenoside Re, a major constituent of ginseng [84], has been shown to activate the PPARγ pathway, resulting in the inhibition of BACE1 at both the transcriptional and translational levels, as well as its activity. This, in turn, leads to the suppression of soluble polypeptide APPβ (sAPPβ) expression and the production of Aβ [85]. Similarly, Notoginsenoside R1 (NTR1), a tetracyclic triterpene present in the root of the popular Chinese medicine Panax ginseng [86], has been shown to inhibit the accumulation of Aβ in APP/PS1 mice and N2a-APP695sw cells, at least in part by activating the PPARγ pathway to increase IDE expression [87]. Meanwhile, it has been reported that ginsenoside Re and NTR1 exhibit improved bioavailability across the BBB when coadministered with Borneol [88]. Astragaloside IV (AS-IV), a tetracyclic triterpene from Astragalus membranaceus [89], has also been shown to be beneficial in neurodegenerative diseases with cognitive impairment [90]. Pharmacokinetic results showed that its elimination half-lives were 98.1, 67.2, and 71.8 min in male rats and 34.0, 66.9, and 131.6 min in female rats at AS-IV doses of 0.75, 1.5, and 3.0 mg/kg, respectively. Moreover, at least some of the AS-IV was able to cross the BBB [91]. AS-IV has been observed to act as a natural agonist for PPARγ, producing results comparable to those achieved by NTR1 in APP/PS1 mice and BACE1-transfected SH-SY5Y cells [92]. Jujuboside A (JuA), a triterpenoid saponin extracted from sour dates [93], has multiple biological activities including neuroprotective properties [94]. In Aβ-induced AD cellular models, JuA activates the Axl receptor tyrosine kinase (Axl)/heat shock protein 90 β (Hsp90β)/PPARγ signaling axis, which restores PPARγ levels and enhances Aβ42 clearance [95]. Finally, ginsenoside Rg1 inhibits the phosphorylation of PPARγ by downregulating the cyclin-dependent kinase (CDK5) pathway and ultimately reduces Aβ levels by affecting the expression of PPARγ target genes, including APP, BACE1, and IDE, in an AD model established using Aβ1-42-treated primary rat neurons [96].

It is noteworthy that certain saponins have been found to have neuroprotective properties, beyond their ability to modulate Aβ metabolism, through the inhibition of Aβ-induced neurotoxicity. For example, hederacolchiside-E, a triterpenoid saponin derived from the Phellodendron plant [97], has been shown to increase neuron activity [98] and prevent Aβ-induced neurotoxicity in both a scopolamine-induced rat AD model and Aβ1-42-induced human neuroblastoma SK-N-SH cells [99]. Similarly, polanoside A from the cactus polaskia chichipe backbg and chikusetsu saponin V, a pentacyclic triterpene saponin, protects SH-SY5Y cells from Aβ-induced cytotoxicity by inhibiting Aβ aggregation [100]. Notably, NTR1 has been found to prevent Aβ-induced neurotoxicity in APP/PS1 mice by increasing the membrane excitability of CA1 pyramidal neurons and reversing Aβ-induced hippocampal long-term potentiation damage [101]. In addition, akebia quinata, commonly known as the chocolate vine, is widely used in traditional medicine throughout East Asia and is also a popular food ingredient [102]. Fluorescence mapping studies have recently revealed the presence of a new pentacyclic triterpene, akequintoside F, mainly extracted from akebia quinata, which shows potential in inhibiting the formation of Aβ-42 plaques [103].

### 2.2. Inhibiting Aberrant Tau Protein Phosphorylation

NFTs, a well-known pathological hallmark of AD [104], are composed of excessively phosphorylated proteins linked by helical filaments known as tubulin-associated unit (tau) protein [105]. Tau protein, mainly found in neuronal axons, is a microtubule-associated protein involved in various functions such as axonal microtubule maintenance, cytoplasmic transport, and regulation of neuronal signaling [106,107]. Its optimal function depends on the low levels of normal phosphorylation [108]. However, overphosphorylation of tau protein leads to its detachment from microtubules, resulting in microtubule depolymerization, aggregation into NFTs, and ultimately neurodegeneration and cell death [109]. The correlation between NFT density and cognitive decline suggests that tau protein plays a central role in AD [110]. Furthermore, the prevalence of tau protein pathology positively correlates with cognitive impairment in AD, where a person with AD has increased levels of tau protein, hyperphosphorylated tau protein, and aggregated tau in their brain [111]. Abnormal tau protein phosphorylation plays a greater role in AD than the total tau protein [112]. As such, it is considered a critical pathogenic step in the development of NFTs and, subsequently, AD [113].

Several saponins have been found to inhibit the hyperphosphorylation of tau proteins, protect synaptic structures, and reduce neuronal damage. An octillol-type saponin, PF11, has been found to increase the activity of phosphatase-2A (PP2A) by enhancing leucine carboxyl methyltransferase-1 (LCMT-1) and improving cognitive impairment in SAMP/8 mice by inhibiting the hyperphosphorylation of tau protein at serine 396 and tyrosine 205 in the brain [63]. Streptozotocin (STZ) is a glucosamine–nitrosourea compound commonly used in AD studies to induce tau proteins’ hyperphosphorylation and cognitive impairment [114]. Tau protein phosphorylation occurs at multiple sites, usually associated with glycogen synthase kinase (GSK) 3 and cyclin-dependent kinase 5 (CDK5). PF11 was also shown to attenuate STZ-induced tau hyperphosphorylation in the brain of male Wistar rats by correcting the dysregulation of the insulin receptor substrate 1/PI3K/Akt/GSK-3β and calpain I/CDK5 signaling pathways [70]. Additionally, PF11 ameliorated STZ-induced learning and memory deficits, synaptic damage, and neuronal death [70]. Another active compound, Ginsenoside Rb1, found in ginseng [115], exhibits neuroprotective properties against different neurotoxins [116]. Research has shown that ginsenoside Rb1 counters cognitive impairment induced by aluminum oxide in ICR mice while reducing Ser396 tau phosphorylation by increasing PP2A levels and decreasing p-GSK levels [111,117]. Ginsenoside Rd, another compound found in ginseng [118], has been shown to have potential therapeutic applications in neurodegenerative diseases [119]. It has been demonstrated to reduce tau proteins’ hyperphosphorylation and neurotoxicity induced by okadaic acid (OA) by increasing PP2A activity in both rat and cortical neuronal models of AD [120].

Theasaponin E1, a vital compound found in green tea seeds, shows remarkable potential in reducing p-tau protein levels and inhibiting AD-promoting genes while activating AD-remitting genes [121]. This potential has been observed in AD models constructed from SHY5Y and glioblastoma (HTB2) cells. Notably, theasaponin E1 successfully reduced p-tau protein by inhibiting several enzymes, including GSK-3β, CDK5, c-Jun NH2-terminal kinase (JNK), MAPK, ERK1/MARK, and calmodulin-dependent protein kinase II alpha [121]. Similarly, Xanthoceraside, a triterpene saponin extracted from the shell of Xanthoceras sorbifolia Bunge, has been shown to exert neuroprotective effects by inhibiting the phosphorylation of tau protein [122]. In a study conducted on mice with AD, the administration of Xanthoceraside significantly decreased the expression of pGSK-3β and tau protein at Ser396 and Ser404, thereby effectively inhibiting the phosphorylation of tau protein [59].

### 2.3. Anti-Inflammatory Effect

In recent years, neuroinflammation has become recognized as a third neuropathological hallmark of AD, in addition to Aβ and NFTs [123]. Neuroinflammation is an inflammatory response to pathological insults that occur within the central nervous system (CNS), such as infection, trauma, ischemia, and toxin accumulation [124]. This response is characterized by the proliferation and activation of microglia and astrocytes in the brain [125]. Such activation is usually accompanied by the secretion of inflammatory cytokines such as interleukin-1β (IL-1β), interleukin 6 (IL-6), and tumor necrosis factor α (TNF-α) [126]. Microglia are innate immune cells that reside within the CNS and are thought to be important regulators in the inflammatory response within the brain [127]. In the early stages of AD, activated microglia can exert protective effects by phagocytosing and degrading Aβ [128,129]. However, when Aβ accumulates excessively, it binds various receptors on the surface of microglia such as cluster of differentiation (CD) 14, CD36 [130], Toll-like receptor (TLR) 4, and TLR2, to promote the release of inflammatory factors [131]. This response leads to neuroinflammation and further impairment of cognitive function. Similarly, astrocyte activation is also associated with Aβ [132]. Activated astrocytes produce various inflammatory mediators [133] and secrete small amounts of Aβ [134], resulting in a chronic vicious cycle [133]. These reactions can exacerbate neuroinflammation and damage neurons.

Multiple pathways are involved in microglia- and astrocyte-mediated neuroinflammation [135], one of which is the nuclear factor kappa-B (NF-κB) pathway [136]. This pathway activates the transcription of TNF-α, IL-1β, and IL-6, which exacerbates inflammation [137]. Microglial activation also depends on other signaling pathways such as the PI3K/Akt/mTOR pathway [138] which can promote the expression of the inflammatory mediators NO synthase (NOS) and cyclooxygenase-2 (Cox-2) [139,140]. Furthermore, MAPK/p38 [141], Janus kinase (JAK)/signal transducer, and activator of transcription (STAT) and other pathways also contribute to neuroinflammation [142]. Therefore, modulating inflammatory signaling pathways and inhibiting the production of proinflammatory factors may offer a promising approach to the treatment of AD.

There is mounting evidence to suggest that certain saponins can effectively inhibit neuroinflammation by blocking the NF-κB pathway. For example, theasaponin E1 has demonstrated anti-inflammatory properties in the treatment of AD by inhibiting the NF-κB pathway in a dose-dependent manner, ultimately leading to a reduction in levels of inflammatory cytokines such as IL-1β, IL-6, and TNF-α [121]. Another example is dioscin, a natural steroidal saponin derived from the rhizomes of dioscoreae nipponicae which has been found to possess a variety of pharmacological activities [143]. In a study involving H_2_O_2_-induced SH-SY5Y cells and an AD C57BL/6J mouse model established with AlCl_3_ and D-gal, dioscin exhibited the ability to inhibit the RAGE/NADPH oxidase 4 (NOX4) signaling axis, leading to a reduction in associated inflammatory factors, such as p-NF-κB/NF-κB, activator protein 1 (AP-1), IL-1β, IL-6, and TNF-α [144]. Additionally, Platycodigenin (PLA), a triterpenoid saponin from Platycodon grandifloras [145], has been shown to have anti-inflammatory and neuroprotective properties [146]. As a potential agonist of the PPARγ pathway, PLA promotes M2 microglial polarization by inhibiting MAPK and NF-κB signaling in Lipopolysaccharide (LPS)-induced BV2 and primary neuronal cells [147]. This leads to a significant reduction in the expression of the proinflammatory cytokines such as TNF-α, Cox2, IL-1β, and inducible nitric oxide synthase (iNOS), while increasing the expression of the anti-inflammatory cytokines such as IL-10, Interleukin-4 (IL-4), mannose receptor, arginase 1 (Arg1), and chitinase-like proteins [147]. As a result, PLA may be an effective modulator of microglial and neurosynaptic activity for the prevention of AD [147]. Furthermore, research has demonstrated that AS-IV possesses potent antineuroinflammatory properties both in vivo and in vitro [148]. Specifically, in experiments conducted on 5xFAD mice and LPS-induced AD models using BV2 cells, AS-IV was found to effectively suppress the activation of the NF-κB pathway and decrease the expression of proinflammatory mediators such as IL-1β, COX-2, iNOS, and TNF-α [148]. These results indicate that AS-IV may be an effective modulator of microglial and neurosynaptic activity for the prevention of AD.

Ginsenoside Rg5, a saponin extracted from ginseng [149], has demonstrated anti-inflammatory properties in BV2 cells by modulating the MAPK and PI3K/Akt pathways, as well as the downstream transcription factors NF-κB and AP-1 [150]. Furthermore, ginsenoside Rg5 has been shown to inhibit LPS-induced NO production and the expression of iNOS, TNF-α, IL-1β, COX-2, and matrix metalloproteinase-9 (MMP-9) in a time-dependent manner [150]. Another compound, CK, has been demonstrated to disrupt NF-κB p65 in Aβ-induced BV2 cells by upregulating low-density lipoprotein receptor-related protein 1 (LRP1) expression, leading to the reduced expression of multiple inflammatory mediators including TNF-α, IL-6, and IL-1β and resulting in a neuroprotective effect 46 [151]. Platycodon grandiflorum is a medicinal herb used in food and medicine [152], of which P. grandiflorum crude saponin (PGS) is its primary constituent with neuroprotective and cognitive-enhancing properties [153]. Notably, PGS has been shown to inhibit the activation of microglial and astrocyte in 5XFAD mice by modulating the NF-κB pathway, thereby reducing neuroinflammation and neuronal loss [153]. Platycodin D (PLD), a triterpene saponin found in Platycodon grandiflorum [145], possesses anticancer, anti-inflammatory, antioxidant, and neuroprotective properties [154]. In cellular assays, PLD has been shown to increase IκBα expressions and reduce TNF-α, IL-1β, and IL-6 levels by inhibiting the TLR4/NF-κB signaling pathway, thereby reducing Aβ-induced inflammation in BV2 cells [155]. Notably, there are pharmacokinetic studies showing that PGS has good BBB permeability, but the ability of Platycodonin D to cross the BBB requires further investigation [156]. Although previous research has suggested that Platycodonin D is able to cross the BBB by comparing its physicochemical parameters with other saponins such as saikosaponin A, glycyrrhizin, and ginsenoside, direct investigation is necessary to confirm its BBB permeability [157]. Moreover, notoginsenoside R2 (NTR2), a bioactive saponin found in Panax ginseng [158], has emerged as a promising treatment for AD [159]. NTR2 has been extensively studied for its ability to reduce COX-2 expression and attenuate Aβ-induced neuroinflammation in primary rat cortical neurons through the microRNA 27a (miR-27a)/SRY-Box Transcription Factor 8 (SOX8)/β-catenin axis [159]. Animal studies in SAMP8 mice have shown that NTR2 treatment or miR-27a antagonism can improve cognitive abilities, highlighting the potential of NTR2 as a treatment for AD [159].

Saponins have been shown to downregulate proinflammatory factors and upregulate anti-inflammatory factors, resulting in the inhibition of microglial or astrocyte activation and preventing neuroinflammation. However, the exact signaling pathways involved in this process are not fully understood. For example, bacopa floribunda (BF), a herb commonly used for memory enhancement and antiaging purposes, has been reported to have therapeutic potential in treating various CNS disorders linked to neuroinflammation [160]. Recent studies have revealed that saponin extracts from BF can inhibit microglial activation and decrease the expression of IL-1β and TNF-α, thereby alleviating neuroinflammation and protecting neuronal cells in the Aβ-induced AD BALB/c mouse model [161]. Additionally, AS-IV has been shown to inhibit the amelioration of Aβ-induced cognitive dysfunction by suppressing neuroinflammation [162]. In an Aβ-induced ICR mouse model of AD, the administration of AS-IV reduced neuroinflammation and neuronal damage by reversing the Aβ-induced increase in TNF-α, IL-1β, and IL-6 levels in a dose-dependent manner [162]. Importantly, ginsenoside Rg1 has also been reported to decrease the expression of NADPH oxidase 2 (NOX2) and NLR family pyrin domain containing 1(NLRP1) inflammasome in H_2_O_2_-induced primary hippocampal neuronal cells from SD rats [163]. The same effect was observed in ginsenoside Rg1-treated APP/PS1 mice, with ginsenoside Rg1 significantly reducing the levels of NLRP1, IL-1β, and TNF-α, alleviating neuroinflammation as well as cognitive dysfunction in the model mice [164]. Such effects hold great promise in the prevention of inflammation and plaque formation, and ultimately lead to the treatment of AD. Moreover, timosaponin BII, the primary constituent of rhizoma Anemarrhenae, a natural remedy used for its anti-inflammatory properties [165]_,_ has been demonstrated to effectively reverse LPS-induced neuroinflammation by reducing the expression of TNF-α and IL-1β in C57BL/6J mice and PC12 cells [166]. Ginsenoside Rb1 has been shown to reverse neuroinflammation in the Aβ ICV injection-induced AD Wistar rat model by reducing the expression of COX-2, IkB-α, neuronal nitric oxide synthase (nNOS), and Aβ1-42 [167]. These findings are similar to the anti-inflammatory effects of PF11 in SD rats with an OA-induced AD model [168], as well as the anti-inflammatory effects of cyclic Cycloastragenol in the Aβ-injected C57BL/6N model [169].

Another natural remedy, ginsenoside Rd, has been shown to alleviate neuroinflammation, reduce neuronal death in the hippocampal region, and enhance learning and memory in an Aβ-induced AD rat model [170]. This effect was achieved by decreasing the expression of ionized calcium-binding adapter molecule 1 (Iba-1), glial fibrillary acidic protein (GFAP), IL1-β, IL-6, and TNF-α, while increasing the expression of IL-10 and heat shock protein 70 (Hsp70) [170]. Another natural remedy with anti-inflammatory properties is Hyoscyamus niger, which has been shown to improve cognitive function and protect against neuroinflammatory damage [171]. Its main constituent, hyoscyamine (Hyo), has been shown to improve cognitive function and protect against neuroinflammatory damage [172]. When taken orally, Hyo reduces the expression of CDK11-P58, suppresses the proinflammatory cytokine IL-6, and increases the production of the anti-inflammatory cytokine IL-4 [173].

In general, natural saponins have anti-inflammatory physiological activity, thereby protecting nerve cells by modulating inflammation-related signaling pathways to inhibit the expression or release of inflammatory factors.

### 2.4. Improvement in Mitochondrial Function and Antioxidative Stress

Oxidative stress refers to an imbalance between oxidation and antioxidation in the body [174], which is mainly characterized by the increased production of reactive oxygen species and reduced ability of antioxidants to combat them [175]. The brain is susceptible to oxidative damage due to its high lipid content and lacks effective antioxidant defense mechanisms, despite high oxygen consumption [176,177]. It is now well established that the level of oxidation in the brain also increases with age [178]. Interestingly, extensive oxidative damage can be observed in the stage of mild cognitive impairment that precedes the typical clinical manifestations of AD, suggesting that oxidative stress may be a central mechanism in driving the disease [179].

Mitochondria, as essential organelles for aerobic metabolism, significantly contribute to the generation of oxidative stress [180]. Meanwhile, it has now been shown that mitochondrial oxidative stress can lead to axonal and neuronal damage and, ultimately, to neurological dysfunction [181]. When oxidative stress occurs within mitochondria, it can lead to mitochondrial swelling, rupture of the outer membrane, and loss of calcium ions, resulting in the further production and release of ROS [182]. Under physiological conditions, ROS can stimulate cell growth [183] and are eliminated by antioxidant enzymes and other antioxidant defense systems [184], such as superoxide dismutase (SOD), glutathione peroxidase (GSH-Px), and catalase (CAT) [185]. However, when a state of redox imbalance occurs, free radicals accumulate in large quantities, leading to lipid peroxidation, resulting in the production of malondialdehyde (MDA) [186], cell membrane damage, mitochondrial membrane damage, and even neuronal death [187]. Furthermore, oxidative stress may interact with Aβ aggregation in the brain [188], tau protein phosphorylation [189], and metal ion microenvironmental damage to amplify neuronal damage, ultimately contributing to the pathogenesis of AD [190,191].

Several natural saponins may act as exogenous antioxidants to combat AD by managing oxidative stress [192,193]. Ginsenoside Rh2, a rare ginsenoside with few reports on its neuroprotective effects compared to other similar molecules [194]_,_ has shown significant neuroprotective effects in a scopolamine-induced model of memory impairment in ICR mice. Ginsenoside Rh2 effectively managed MDA levels while increasing glutathione (GSH) levels and SOD activity in the brain, effectively inhibiting oxidative stress [195]. These findings suggest that ginsenoside Rh2 has great potential for the treatment of AD by inhibiting oxidative stress in the brain. Crude saponins from BF showed potent antioxidant effects in addition to anti-inflammatory effects. In addition, saponins from BF alleviated oxidative stress, reduced ROS, and MDA levels by restoring GSH-Px, GSH, and CAT levels in the Aβ ICV-induced BALB/c AD mouse model [161]. Similarly, ginsenoside Rd was found to reduce protein carbonyls and 4-hydroxy-2-nonenal (4-HNE) levels by decreasing the glutathione disulfide (GSSG)/GSH ratio, thereby counteracting oxidative stress in the brain of Aβ-induced AD mouse models [170]. Ginsenoside Rg3, a tetracyclic triterpenoid saponin, is the major bioactive component of ginseng and is widely used for the treatment of tumors [196]. Results from in vivo studies have shown that ginsenoside Rg3 can improve mitochondrial dysfunction and enhance ROS scavenging ability by increasing SOD, CAT, and GSH-Px levels, thus ameliorating oxidative stress in the brain of D-gal-induced AD rats [197]. PF11 was found to inhibit the production of both APP and Aβ in the cortex and hippocampus, while restoring the activity of SOD and GSH-Px in the brains of AD mice and reducing MDA levels in the cortex [198].

Ginsenoside Rg1 has also been found to protect primary cortical neurons against Aβ-induced increases in ROS and H_2_O_2_ levels by inhibiting oxidative stress [199]. This results in a decrease in mitochondrial membrane potential and an increase in cytochrome C (Cyt-c) oxidase activity, ultimately leading to a decrease in Cyt-c release and indicating improved mitochondrial function [199]. Another newly discovered saponin compound, huangjingsterol B from Polygonatum cyrtonema Hua, showed significant antioxidant activity [200] by reducing oxidative stress and protecting cells from Aβ-induced damage through modulating the activity of GSH-Px and SOD [200]. Similarly, researchers have found that a saponin from the fruit of Solanum anguivi can protect the P2 region of rat brain synaptosomes from oxidative damage induced by ferrous iron and sodium nitroprusside, reduce ROS generation, restore total thiol levels, and protect mitochondrial function [192]. This suggests that saponins from Solanum anguivi fruits may have antioxidant properties that benefit mitochondrial function [201]. PGS has a similar effect with its ability to upregulate intracellular heme oxygenase-1 (HO-1), SOD, CAT, and GSH-Px to inhibit Aβ-induced ROS production and reduce oxidative damage in AD brains [153]. Despite the demonstrated neuroprotective effects of Panax ginseng, the mechanisms underlying its antioxidant properties remain poorly understood. However, a recent study has shown that the primary bioactive compound in Panax ginseng, NTR1, is able to prevent the loss of mitochondrial membrane potential and reduce the accumulation of ROS induced by Aβ [202]. This suggests that NTR1 may exert neuroprotective effects by inhibiting oxidative stress. Furthermore, most previous experiments have focused on saponins extracted from Panax ginseng roots, and the pharmacological effects of other parts, such as saponins in P. notoginseng leaves (LPNS), are largely unknown [203]. LPNS, which mainly include the ginsenosides Rb1, Rb2, Rb3, and Rc, have been shown to have potent neuroprotective properties [203]. In H_2_O_2_- or oxygen and glucose deprivation (OGD)-induced astrocytes from the cortex of SD rats and SH-SY5Y cells, LPNS increased the levels of nuclear factor erythroid2-related factor 2 (Nrf2) and its downstream HO-1 and glutathione S-transferase pi 1 (GSTP1) in response to increased ROS, as well as significantly reducing lactate dehydrogenase (LDH) expression and increasing the cellular activity of astrocytes [203].

In other studies, saponins have been shown to alleviate oxidative stress by modulating oxidative stress-related pathways. In an Aβ-induced AD model, treatment with Cycloastragenol resulted in elevated levels of Nrf2 and HO-1 and a decrease in levels of ROS and lipid peroxidation (LPO) in the mouse brain, highlighting its potent antioxidant activity [169]. Similarly, Ginsenoside Rg1 responded to H_2_O_2_-induced PC12 cell injury by downregulating the NF-κB signaling pathway, as well as its upstream Akt and ERK1/2, thereby increasing the expression of inhibitor kappa B alpha (IκB-α) by inhibiting the phosphorylation of NF-κB/p65 and IκB-α [204]. Additionally, Dioscintn was found to improve physical coordination and enhance learning memory and spatial exploration in a mouse model by modulating the RAGE/NOX4 signaling axis, upregulating the expression of Nrf2, HO-1, and SOD in the oxidative stress pathway, and inhibiting the production of MDA and ROS [144]. Furthermore, recent research indicates that ginsenoside Rg1 enhances the antioxidant activity of SOD, CAT, and GSH-Px by modulating the wingless (Wnt)/GSK-3β/β-catenin signaling pathway [205], which leads to a decrease in oxidative products such as nitrotyrosine, 8-hydroxyguanosine (8-OHG), and MDA. This ameliorated oxidative stress damage, protected neurons, and ameliorated cognitive impairment in AD tree shrews [205]. Esculentoside A, a saponin from the roots of phytolaca esculenta [206], has been shown to reduce oxidative stress in 3 × Tg-AD mice [207]. This compound activates PPARγ, which decreases MDA and ROS and increases GSH and SOD levels, with its effects reversed by the PPARγ inhibitor GW9662 [207]. Additionally, in Aβ-induced PC12 cells, ginsenoside Rb1 can act as a PPARγ agonist to promote cholesterol efflux, thereby attenuating Aβ-induced cholesterol elevation, ROS accumulation and lipid peroxidation, and, finally, reducing cell death [208]. Furthermore, PLD was found to attenuate Aβ-induced oxidative stress in BV-2 cells by activating the Nrf2/HO-1 pathway [155]. This was evidenced by a reduction in ROS and MDA production, as well as an increase in SOD and NQO1 activity [155]. Furthermore, the antioxidant effect of PLD in Aβ-stimulated BV-2 cells was abolished by transfection with si-Nrf2, suggesting that the antioxidant activity of PLD is closely linked to the Nrf2/HO-1 signaling pathway [155]. Similarly, ginsenoside Re increased the expression of NAD (P) H dehydrogenase 1 and glutamate-cysteine through the activation of the Nrf/HO-1 pathway, as well as GSH levels, and the activities of SOD, glutamate-cysteine ligase regulatory subunit (GCLC) and GSH-Px [209]. These effects effectively reversed Aβ-induced ROS production in SH-SY5Y cells, thereby protecting them from oxidative stress damage [209]. Saikosaponin-D, the main component of Bupleurum falcatum [210], has been shown to cross the BBB directly to exert neuroprotective effects [211]. It has now been found that in glutamate-induced SH-SY5Y, Saikosaponin-D increases the activity of several endogenous antioxidant enzymes and the expression of Nrf2 and HO-1 through the activation of the PI3K pathway, thereby making SH-SY5Y cells free from glutamate neurotoxicity [212]. These findings demonstrate the potential of these compounds as effective antioxidants for the treatment of AD.

### 2.5. Antiapoptotic Effect

It is widely accepted that neuronal apoptosis contributes significantly to the pathogenesis of AD [213] and is even a pathological hallmark of the disease [214]. Neuronal apoptosis has been found to be abundant in AD autopsy samples [215] and several AD models have successfully reproduced this apoptosis [216]. The biochemical signature of apoptosis is mainly the activation of cysteinase triggered by intrinsic or extrinsic apoptotic pathways [217]. Apoptosis triggered by external cellular signals is called the “death receptor“ pathway, which involves the activation of caspase-8 followed by cleavage of caspase-3, leading to apoptosis [218]. In contrast, the intrinsic apoptotic pathway is driven by the mitochondria [219]. Mitochondrial damage induces the release of Cyt-c and other apoptotic factors into the cytoplasm, which then activate caspase-9 and caspase-3, resulting in apoptosis [220]. The B-cell lymphoma-2 (Bcl-2) protein family includes proapoptotic proteins, BCL2-associated X (BAX), Bcl-2 antagonist killer 1 (BAK), Bcl-2-like protein 11 (BIM), p53 upregulated modulator of apoptosis (PUMA), and Bcl2 modifying factor (BMF), and antiapoptotic proteins, such as Bcl-2, lymphoma-extra-large (Bcl-xL), Bcl-2 like 2 (Bcl-w), and myeloid cell leukemia-1 (Mcl-1), which tightly regulates this apoptotic process by controlling the ratio of proapoptotic and antiapoptotic proteins [221,222], particularly the Bax/Bcl-2 ratio [223]. In AD patients, Bcl-2 expression is reduced in the hippocampus [224], while Bax accumulates near senile plaques and tau protein fiber tangles in an AD animal model, suggesting that mitochondria-mediated apoptosis is closely linked to the pathology of AD [225].

Numerous saponins, including astragaloside (AST), NTR2, PGS, AS-IV, NTR1, Ginsenoside Rg2, and Ginsenoside Rg1, have shown antiapoptotic effects in AD-associated models. AST, a small molecule saponin derived from Astragalus membranaceus, has been widely used in China for the treatment of chronic diseases [226]. In in vivo and in vitro experiments, AST can prevent Aβ-induced apoptosis in cortical neurons by modulating the PI3K/Akt and MAPK (or ERK) pathway [227]. This effect is reversed by the PI3K inhibitor LY294002 and mimicked by the inhibition of ERK and enhanced by its inhibitor U0126, suggesting that the neuroprotective effect of AST may also be related to the inhibition of the ERK pathways [227]. Similarly, NTR2 can reverse the Aβ-induced apoptosis of primary cortical neurons via miR-27a/SOX8/axis and ameliorate cognitive dysfunction in AD mice [159]. PGS may provide neuroprotection not only by reducing inflammation but also by inhibiting apoptosis. PGS increased the expression of Bcl-2 family proteins, resulting in a reduction in Cyt-c release and caspase-9 and -3 expression, thereby inhibiting Aβ-induced apoptosis in HT22 cells. PGS achieved this by inhibiting p38, ERK, and JNK activation, as evidenced by decreased levels of p-p38/p38, p-ERK/ERK, and p-JNK/JNK, and by downregulating MAPK signaling [153]. In vitro and in vivo experiments constructed in HT-22 cells and C57BL/6 induced by amyloid beta protein fragment 1-42 oligomers (AβO) have shown that AS-IV reduced the Aβ-induced inhibition of the brain-derived neurotrophic factor (BDNF)–tyrosine kinase receptor B (TrkB) signaling pathway by regulating PPARγ [228]. This mechanism leads to the reduction in the number and fluorescence density of terminal deoxynucleotidyl transferase dUTP nick end labeling (TUNEL)-positive cells and caspase-3 levels, ultimately alleviating hippocampal neuronal apoptosis and memory impairment [228]. In Aβ-induced PC12 cells, NTR1 has also been shown to exert an antiapoptotic effect, reducing the percentage of TUNEL-positive cells and the levels of caspase-3, Bax, and the Bax/Bcl-2 ratio, and exerted an antiapoptotic effect by suppressing the MAPK pathway [202].

Ginsenoside Rg2 and Rg1 have been found to inhibit apoptosis and improve cognitive function in AD rat models through different mechanisms. In the Aβ-induced SD rat model of AD, ginsenoside Rg2 increased the Bcl-2/Bax ratio and reduced caspase-3 activity by activating the PI3K/Akt signaling pathway, thereby reducing Aβ-induced apoptosis and improving cognitive function in a rat model of AD [229]. Ginsenoside Rg1 reduced the number of TUNEL-positive cells in the brains of AD rats, decreased the expression of glucose-regulated protein 78, and inhibited the ER stress-mediated apoptotic pathway of c-JNK [230]. Additionally, Ginsenoside Rg1 treatment reduced the expression of tumor necrosis factor receptor-associated factor 2 and inositol-requiring enzyme-1 and inhibited the activation of p-JNK. These findings suggest that Ginsenoside Rg1 has neuroprotective effects in AD rats [230]. Acori graminei Rhizoma (AGR) is a TCM used for AD with fewer side effects, but the underlying mechanism is unclear [231]. Recently, both in vivo and in vitro experiments have demonstrated that the combination of AGR and ginsenoside Rg1 is able to suppress HO-1 expression by increasing miR-873-5p expression, thereby attenuating miR-873-5p-mediated neuronal cell apoptosis and the cognitive dysfunction in mice with AD [232].

There are natural saponins that can function as natural inhibitors of apoptosis by regulating apoptosis-related targets directly. In an Aβ-induced AD rat model, pretreatment with ginsenoside Rd reduced the expression of caspase-3, suggesting both antiapoptotic and then neuroprotective effects [170]. Furthermore, ginsenoside Rg3 has been shown to increase the expression of Bcl-2 by reversing the abnormal increase in caspase-3, caspase-9, Bax, apoptosis-inducing factor (AIF), and Cyt-C induced by D-gal in AD models [197]. This resulted in a reduction in the number of TUNEL-positive cells, inhibition of D-gal-mediated apoptosis, and prevention of cognitive impairment in AD rats [197]. Similarly, PF11 significantly attenuated the expression of JNK 2, p53, and cleaved caspase-3 in the brains of APP/PS1 mice and Aβ-induced KM mice [198]. This suggests that the beneficial effects of PF11 on neuronal function may be due to its antiapoptotic properties, in addition to its previously mentioned inhibition of Aβ production [198]. Treatment with Cycloastragenol improved the learning and memory abilities of Aβ-induced mice, which was closely related to its reduction in Bax, caspase-3, and BIM expression and increased Bcl-2 expression in the brains of Aβ-induced mice [169]. Lychee seed is a TCM known to regulate blood sugar and lower blood lipids, as well as prevent liver damage, and have antioxidant, antiviral, and anticancer properties [233]. Recently, the saponin extract of lychee seed (LSS) was found to alleviate cognitive dysfunction by inhibiting cell apoptosis. In both Aβ-induced PC12 cells and AD rats, LSS can reduce the expression of homocysteine-3 and Bax, while increasing the expression of Bcl-2 and the ratio of Bcl-2/Bax, reversing apoptosis, and, ultimately, alleviating cognitive dysfunction in AD rats [234]. Polygalasaponin F (PGSF), a triterpenoid saponin monomer isolated from P. japonica, has been shown to improve hippocampal-dependent learning memory capacity [235]. Furthermore, it has also been reported to reduce the number of caspase3-positive cells, thereby reversing glutamate-induced apoptosis in primary hippocampal neurons [236].

## 3. Discussion

The summarized saponins, as shown in Table 1, are primarily categorized into monomeric saponins and total saponin extracts. Based on the sapogenin skeletons, monomeric saponins used for AD treatment can be divided into two categories: triterpenoid saponins and steroidal saponins. Triterpenoid saponins are the most common, with the majority derived from pentacyclic triterpenoids (oleanane) and tetracyclic triterpenoids (dammarane). On the other hand, most steroidal saponins are derived from spirostane. Accordingly, triterpenoid saponins and sapogenin skeletons such as oleanane, dammarane, and spirostane play a key role in AD treatment.

When it comes to model selection, in vivo studies mostly rely on AD models induced by Aβ or transgenic mice such as SAMP/8 and APP/PS1, while in vitro studies usually employ Aβ-induced primary neuronal cells, PC12 cells, and SH-SY5Y cells. The duration of saponin administration ranges from 1 to 72 h for in vitro experiments and typically from 4 days to 3 months for in vivo experiments. This selection of models and duration of treatment is essential in ensuring the efficacy and safety of saponin-based therapies for AD, and the results of these studies can provide valuable insight into the potential effectiveness of saponins in clinical settings.

**Figure 2:**
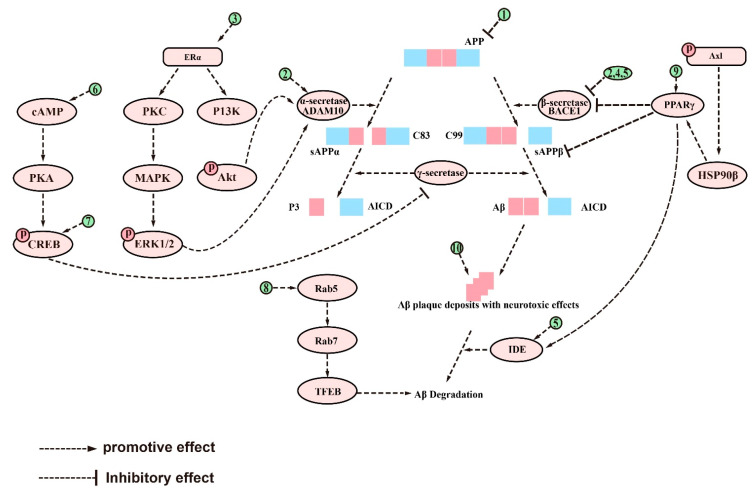
The signaling pathways and targets of Aβ metabolism regulation by saponins. (1). Ginsenoside Rg1 and xanthoperidinol, to inhibit Aβ formation by reducing APP production; (2). Ginsenoside Rg1 and theasaponin E1, can enhance α-secretase activity and decrease β-secretase and γ-secretase activity; (3). Ginsenoside Rg1, regulates targets of PKC, MAPK, and PI3Ksignaling pathways; (4). RAPO-1-3, onjisaponin B, and PF11, can decrease β-secretase activity; (5). CK, can reduce the expression of BACE1 and PS1 and increase the activity of IDE; (6). Ginsenoside Rg1, regulates targets of PKA/pCREB signaling pathways; (7). Minor ginsenoside F1, can enhance the expression of pCREB; (8). PF11, can recover Rab conversions; (9). Ginsenoside Re, NTR1, AS-IV, Jujuboside A, and ginsenoside Rg1, regulate targets of PPARγ signaling pathways; (10). Hederacolchiside-E, polanoside A, chikusetsu saponin V, NTR1, and Akquintoside F, can directly inhibit the neurotoxicity of Aβ. The above-mentioned saponins inhibit the metabolism of Aβ by modulating the targets of these signaling pathways.

**Figure 3:**
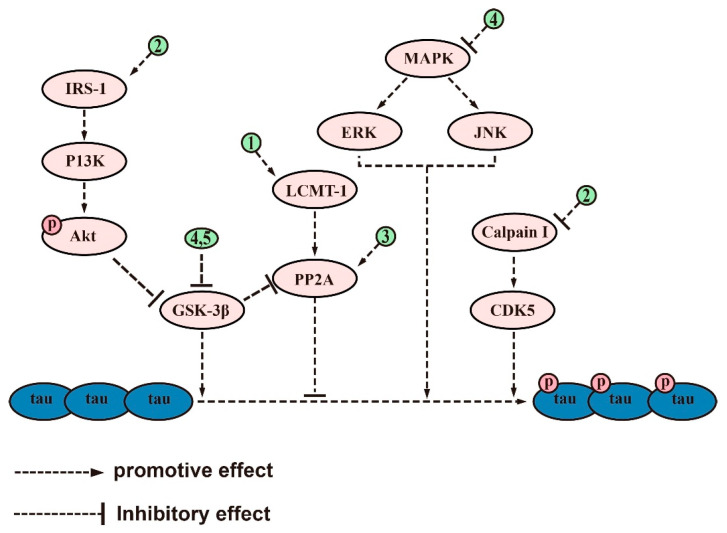
The signaling pathways and targets of saponin-regulated tau protein phosphorylation. (1). PF11 and xanthoperidinol, regulate targets of LCMT-1/PP2A signaling pathways; (2). PF11, regulates targets of IRS-1/P13K/Akt/GSK-3β and calpain I/CDK5 signaling pathways; (3). Ginsenoside Rb1 and Ginsenoside Rd, regulate the activity of PP2A; (4). Theasaponin E1, inhibits tau protein phosphorylation by regulating GSK-3β, CDK5, c-Jun JNK, MAPK, ERK1/MARK, and PP2A; (5). Xanthoceraside, regulates the activity of GSK-3β. The above-mentioned saponins inhibit tau protein phosphorylation by modulating the targets of these signaling pathways.

**Figure 4:**
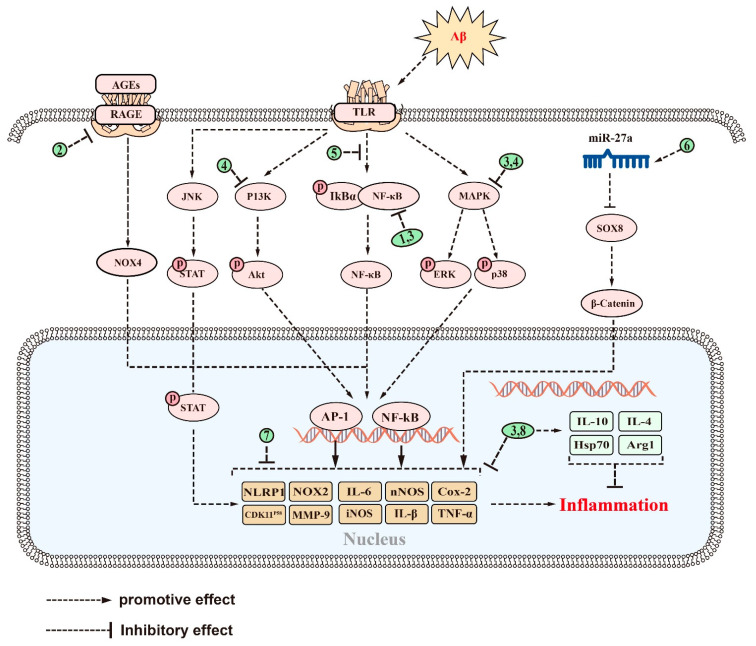
The signaling pathways and targets of saponins against neuroinflammation in AD. (1). Theasaponin E1, CK, PGS, and AS-IV, regulate targets of NF-κB signaling pathway; (2). Dioscin, regulates targets of RAGE/NOX4 signaling pathway; (3). Platycodigenin, regulates targets of MAPK and NF-κB signaling pathways; (4). Ginsenoside Rg5, regulates targets of MAPK and PI3K/Akt signaling pathways; (5). PLD, regulates targets of TLR4/NF-κB signaling pathway; (6). NTR2, regulates targets of miR-27a/SOX8/β-catenin signaling pathways; (7). BF, AS-IV, Ginsenoside Rg1, Timosaponin BII, ginsenoside Rb1, and PF11, regulate various inflammatory factors, such as IL-16, IL-1β, etc.; (8). Ginsenosides Rd and hyo, enhance the expression of many anti-inflammatory factors, such as IL-10 and Hsp70, in addition to reducing inflammatory factors. The above saponins suppress neuroinflammation in AD through regulating targets of these signaling pathways.

**Figure 5:**
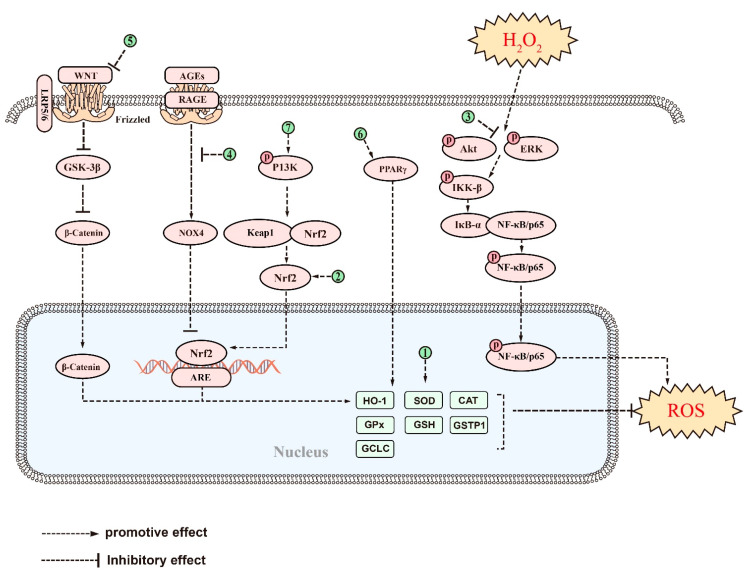
The signaling pathways and targets of saponins against oxidative stress in AD. (1). Ginsenoside Rh2, Crude saponins from BF, ginsenoside Rd, ginsenoside Rg3, PF11, ginsenoside Rg1, huangjingsterol B, saponin from the fruit of Solanum anguivi, and PGS, increased expression of many antioxidant factors such as GSH-Px, SOD, CAT, etc.; (2). LPNS, Cycloastragenol, PLD, and ginsenoside Re, regulate targets of Nrf2/HO-1 signaling pathway; (3). Ginsenoside Rg1, regulates targets of NF-κB signaling pathways; (4). Dioscintn, regulates targets of RAGE/NOX4signaling pathways; (5). Ginsenoside Rg1, regulates targets of Wnt/GSK-3β/β-catenin signaling pathway; (6). Esculentoside A and Ginsenoside Rb1, regulate targets of PPARγ signaling pathways; (7). Saikosaponin-D, regulates targets of PI3Ksignaling pathway.T above saponins suppress oxidative stress in AD through regulating targets of these signaling pathways.

**Figure 6:**
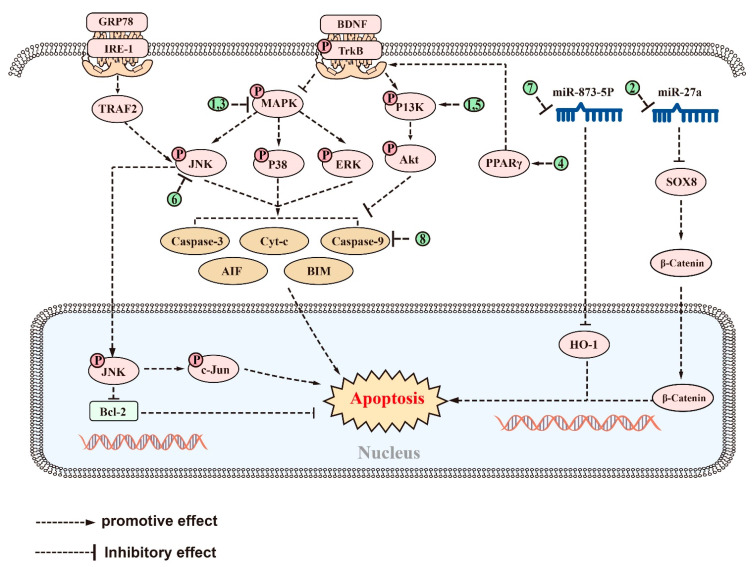
The signaling pathways and targets of saponins against apoptosis in AD. (1). AST, regulates targets of PI3K/Akt and MAPK (orERK) signaling pathways; (2). NTR2, regulates targets of miR-27a/SOX8 signaling pathway; (3). PGS and NTR1, regulate targets of MAPK signaling pathways; (4). AS-IV, regulates targets of PPARγ/BDNF signaling pathways; (5). Ginsenoside Rg2, regulates targets of PI3K/Akt signaling pathway; (6). Ginsenoside Rg1, inhibits the activation of p-JNK; (7). AGR, regulates targets of miR-873-5p signaling pathways; (8). Ginsenosides Rd, ginsenoside Rg3, PF11, Cycloastragenol, LSS, and PGSF, can directly regulate apoptosis-related targets such as caspase-3, Bax, and AIF, etc. The above saponins suppress apoptosis in AD through regulating targets of these signaling pathways.

In terms of efficacy, saponins have been found to alleviate the symptoms of AD by inhibiting Aβ accumulation, aberrant tau phosphorylation, neuroinflammation, oxidative stress, and apoptosis. The anti-Aβ deposition effects of saponins, including ginsenoside Rg1, RAPO-1-3, and onjisaponin B, were achieved by regulating APP processing. They also inhibit Aβ neurotoxicity, demonstrating their good neuroprotective effects. Additionally, saponins such as PF11, Theasaponin E1, and Xanthoceraside can alleviate neurotoxicity caused by tau overphosphorylation, protecting neuronal cells from damage. Saponins also exhibit anti-inflammatory effects, with examples such as theasaponin E1, dioscin, and PLA working to reduce the levels of proinflammatory cytokines such as TNF-α, IL-1β, IL-6, Cox2, iNOS, and MMP-9 while promoting the levels of anti-inflammatory cytokines IL-10 and Arg1 by regulating NF-κB, TLR4/NF-κB, and the miR-27a/SOX8/β-catenin, RAGE/NOX4, MAPK, PPARγ, and PI3K/Akt signaling pathways. Furthermore, saponins such as ginsenoside Rh2, crude saponins from BF, and ginsenoside Rd exhibit antioxidant properties by increasing neuronal activity and reducing the levels of ROS, MDA, NO, iNOS, and NOX4 by regulating the NF-κB, Akt/Nrf2, ERK1/2, RAGE/NOX4, Wnt/GSK-3β/β-linked protein, PPARγ, and Nrf2/HO-1 signaling pathways while increasing the activity of SOD, CAT, GPx, GSH, and GSH-Px. Finally, saponins such as AST, NTR2, and PGS are known for their antiapoptotic role by inhibiting the levels of proapoptotic proteins such as Bax, Cyt-c, caspase-8, and caspase-3 while increasing the level of Bcl-2. They achieve this by regulating the PI3K/Akt, MAPK, ERK, miR-27a/SOX8, MAPK, PPARγ, BDNF-TrkB, and mitochondrial apoptotic pathways. Figure 2, Figure 3, Figure 4, Figure 5 and Figure 6 provide a comprehensive overview of the signaling pathways and targets of saponin drugs against AD, indicating that different saponins act on similar signaling pathway targets to treat AD, while the same saponin drugs act on multiple targets across different signaling pathways. Collectively, saponins have been shown to have a positive impact on various mechanisms associated with the onset and progression of AD. However, their consistent effect is the enhancement of neuronal cell activity, leading to improved learning and memory abilities in experimental animals and ultimately alleviating cognitive dysfunction. Notably, in addition to invasive and pharmacological therapies, a number of noninvasive, nonpharmaceutical therapies are now demonstrating very high potential in the treatment of neurological disorders [237]. In clinical practice, a combination of pharmacological treatment and noninvasive brain stimulation techniques has been reported to modulate brain activity during the consolidation and fading of fear memories [238,239]. These findings not only deepen our understanding of AD but also provide new avenues for the treatment of AD.

Taken together, saponins represent a promising class of natural compounds with neuroprotective effects found in various plants, showing considerable potential in the prevention and treatment of AD. In contrast to drugs that elevate acetylcholine levels, saponins are able to exert positive therapeutic effects by inhibiting Aβ accumulation and tau protein phosphorylation, reducing oxidative stress and inflammation, and decreasing apoptosis. Furthermore, their natural origin makes them readily available and generally safe, albeit occasional side effects have been reported. For example, some animal studies have found that saponins can interact with cell membranes and form pores, which can destabilize cell membranes [240] and cause hemolytic toxicity, leading to anemia [241]. Secondly, long-term use of ginsenosides can lead to estrogen-like effects, resulting in symptoms such as vaginal bleeding and breast pain in some patients [242]. In addition, it should be noted that taking ginsenosides in large quantities for extended periods can lead to insomnia and high blood pressure [243]. At the same time, ginsenosides can also cause abdominal pain, diarrhea, and vomiting due to potential intestinal wall irritation and damage [244]. Furthermore, there is some evidence indicating that high doses of saikosaponins can lead to increased serum transaminase activity and liver SOD activity in mice and rats, leading to liver damage [245]. Despite these potential side effects, their great promise as a treatment for AD remains unchanged, although more research is needed to fully understand the safety and efficacy of saponins for the treatment of AD.

## 4. Conclusions and Perspective

Current studies suggest that saponins, including both triterpenoid and steroidal saponins, have the potential to treat AD by reducing Aβ deposition, inhibiting tau protein phosphorylation, regulating oxidative stress, reducing inflammation, and promoting neuronal survival. The detailed signaling pathways through which saponins exert their effects have been described above and in Figure 2, Figure 3, Figure 4, Figure 5 and Figure 6. However, there are several issues that require further investigation. Firstly, most of the studies have been carried out on animals or cells, whereas human clinical trials have been rare. Secondly, most of the saponins are derived from natural drugs, and their extraction or isolation presents some difficulties [246]. Thirdly, although natural saponins are effective, future chemical modifications and synthesizing may improve their activity and yield while reducing adverse effects [247]. Despite these challenges, saponins hold potential as therapeutic agents for the treatment of AD, but further studies are necessary to fully comprehend their mechanisms and potential benefits.

## 5. Limitations and Future Directions

Every research project has limitations, and this review is no exception. Therefore, there are several issues which need to be addressed in further studies. Firstly, the saponins discussed in this review for AD treatment are mostly total saponins, rather than specific individual components. Thus, further exploration of saponins’ activity, isolation of their individual components, and elucidation of their mechanisms of action are necessary. Secondly, although numerous animal or cellular studies have been conducted, data from human clinical trials are scarce. Future studies must focus on clinical trials to better understand the therapeutic effects and side effects of saponins. Furthermore, there have been few pharmacokinetic studies on saponins, limited information on their blood–brain barrier permeability and plasma half-life. Therefore, in future research, more attention should be paid to the study of pharmacokinetics. Finally, current studies have overlooked the synergistic effects between saponins. Although this is very challenging, further research should focus on investigating the deeper mechanisms of the synergistic effects between saponins to better understand AD.

## Figures and Tables

**Table 1:** Saponins against AD. The table covers all the saponins that appear in this review. The specific information includes the saponin name, source, dose, and time of administration, mechanism, Specific Targets, pathway, and the location of its corresponding reference in this paper. Up arrows indicate upregulation, while the down arrows indicate downregulation.

Compound and Original Source	Model	Dose	Drug Administration Time	Mechanism	Target	Signaling Pathway	Refs.
Ginsenoside Rg1, ginseng	Male APP/PS1 mice	0.1, 1, 10 mg/kg	30 days	Inhibition of Aβ deposition	APP, Aβ↓	Unknown	[56]
Xanthoperidinol, husks of xanthoceras sorbifolia bunge	Male APP transgenic mice	0.02, 0.08 or 0.32 mg/kg	6 months	Inhibition of Aβ deposition	APP, Aβ↓	Unknown	[59]
Ginsenoside Rh2, ginseng	Tg2576 mice	10 mg/kg	8 weeks	Inhibition of Aβ deposition	APP, Aβ↓ sAPPα↑	Unknown	[60]
Ginsenoside Rg1, ginseng	Male Tg mapp mice	10 mg/kg	3 months	Inhibition of Aβ deposition	Aβ, γ-secretase↓	Unknown	[61]
Ginsenoside Rg1, ginseng	OVX and d-gal-induced female Wistar rats	5, 10, 20 mg/kg	6 weeks	Inhibition of Aβ deposition	Aβ, BACE1↓ ADAM10↑	Unknown	[67]
Ginsenoside Rg1, ginseng	HT22 cells and SH-SY5Y cells stably expressing the Swedish mutant APP Female Wistar rats	In vitro: 2–10 μM In vivo: 10 mg/kg	In vitro: 30 min In vivo: 8 weeks	Inhibition of Aβ deposition	Aβ, sappα↓ α-secretase↑	ERK/MAPK PI3K/Akt	[68]
RAPO-1-3 and onjisaponin B, radix polygalae	Male APP/PS1 mice	In vivo: 200 μL of Onjisaponin B (1 mg/mL), RAPO-1-3 (15 mg/mL)	3 months	Inhibition of Aβ deposition	Aβ, APP↓ Interfered with the interaction between PS1 and BACE1	Unknown	[62]
Pseudoginsenoside-F11, ginseng	Male SAMP8 mice	2, 8, 32 mg/kg	3 months	Inhibition of Aβ deposition	Aβ, BACE1↓	Unknown	[63]
Theasaponin E1, green tea seeds	Sweapp N2a cells	5, 10, 15, 20, 25, and 30 μg/mL	24 h	Inhibition of Aβ deposition	Aβ, γ-secretase, BACE1, APP↓ Neprilysin, ADAM10↑	Unknown	[64]
Ginsenoside compound K Protopanaxadiol saponin, panax notoginseng	Scopolamine-induced male ICR mice	20, 40 mg/kg	14 days	Inhibition of Aβ deposition	BACE1, PS1, Aβ↓ IDE↑	Unknown	[66]
ginsenoside (20S)-Rg3, ginseng	Primary neuron APP/PS1 mice	In vitro: 10, 30, 50 μM In vivo: 20 mg/kg	In vitro: 6 h In vivo: 10 weeks	Inhibition of Aβ deposition	Aβ, γ-secretase↓ PI4KIIα↑	Unknown	[65]
Minor ginsenoside F1, ginseng	Male APP/PS1 mice	20 mg/kg	8 weeks	Inhibition of Aβ deposition	Aβ↓	Unknown	[75]
Bacopaside I, bacopa monniera	Male APP/PS1 mice	15, 50 mg/kg	2 months	Inhibition of Aβ deposition	Aβ↓ Innate immune stimulation and phagocytosis	Unknown	[79]
Pseudoginsenoside-F11, ginseng	Primary rat microglial cells	10, 30, and 100 μM	6 h	Inhibition of Aβ deposition	Aβ↓ Cate, LAMP2, V-ATP, Rab5 and Rab7↑	Mtor/TFEB	[82]
Ginsenoside Re, ginseng	The n2a/APP695 cell line that stably expressed Swedish mutant human APP695	0, 25, 50, 100, 150, and 200 μM	24 h	Inhibition of Aβ deposition	Sappβ, BACE1, Aβ↓ PPARγ↑	PPARγ	[84]
Notoginsenoside R1, panax notoginseng	N2a-APP695sw cells Male APP/PS1 mice	In vitro: 1, 10, 100 μM In vivo: 5, 25 mg/kg	In vitro: 48 h In vivo: 3 months	Inhibition of Aβ deposition	Aβ↓ PPARγ, IDE↑	PPARγ	[84]
Astragaloside IV, astmgali Radix	SH-SY5Y cells transfected with BACE1 Male APP/PS1 mice	In vitro: 250 μg/mL In vivo: 1, 10 mg/kg	In vitro: 24 h In vivo: 3 months	Inhibition of Aβ deposition	BACE1, Aβ↓ PPARγ↑	PPARγ	[92]
Jujuboside A, semen ziziphi spinosae	Aβ-induced BV2 cells Male APP/PS1 mice	In vitro: 1, 5, 25 μM In vivo: 0.5, 1.5, or 5 mg/kg	In vitro: 18 h In vivo: 7 days	Inhibition of Aβ deposition	Aβ↓ PPARγ, Axl, HSP90β↑	Axl/HSP90/PPARγ	[95]
Ginsenoside Rg1, ginseng	Aβ-induced rat hippocampal neurons	60 µM	1 h	Inhibition of Aβ deposition	CDK5, BACE1, APP and Aβ↓ IDE↑	PPARγ	[96]
Hederacochiside-E, pulsatilla koreana	Aβ-induced human neuroblastoma SK-N-SH cells Scopolamine-induced rats	In vitro: 1, 10, 30, 100 μM In vivo: 30, 60 mg/kg	In vitro: 48 h In vivo: Unknown	Inhibition of Aβ neurotoxicity	Neurotoxicity of Aβ↓ Neuronal cell activity↑	Unknown	[99]
Polanoside A and chikusetsusaponinv, polaskia chichipe backbg	SH-SY5Y cells	25, 50 μM	24 h	Inhibition of Aβ neurotoxicity	Neurotoxicity of Aβ↓	Unknown	[100]
Notoginsenoside R1, panax notoginseng	C57BL/6J mice and APP/PS1 mice	5 mg kg	4 months	Inhibition of Aβ neurotoxicity	Neuronal excitability↑	Unknown	[101]
Akequintoside F, akebia quinata	Aβ-induced E. Coli	40 μM	6 h	Inhibition of Aβ neurotoxicity	Inhibited Aβ-induced fibrosis	Unknown	[103]
Pseudoginsenoside-F11, ginseng	Male SAMP8 mice	2, 8, 32 mg/kg	3 months	Inhibiting aberrant tau-like protein phosphorylation	Tau hyperphosphorylation in serine 396 and tyrosine 205↓ m-PP2A, LCMT-1↑	Unknown	[63]
Pseudoginsenoside-F11, ginseng	STZ-induced male Wistar rats	2, 4, 8 mg/kg	4 weeks	Inhibiting aberrant tau-like protein phosphorylation	The autolytic form of calpain, p35, p85, p25, CDK5, Hyperphosphorylation of tau protein at the specific sites Ser396 and Ser199/202 ↓ P-GSK-3β, phosphorylated AKT↑	Calpaini/CDK5	[70]
Ginsenoside Rb1, ginseng	Alcl_3_-induced 32 female ICR mice	20 mg/kg	4 months	Inhibiting aberrant tau-like protein phosphorylation	Hyperphosphorylation of tau protein at the specific sites Ser396, p-GSK3 ↓ PP2A↑	Unknown	[117]
Ginsenoside Rd, ginseng	Cortical neurons cells OA-induced Sprague Dawley rats	In vitro: 2.5, 5 μM In vivo: 10 mg/kg	In vitro: 12 h In vivo: 7 days	Inhibiting aberrant tau-like protein phosphorylation	Hyperphosphorylation of tau protein at ps199/S202 and ps396↓ PP2A↑	Unknown	[120]
Theasaponin E1, green tea seeds	SH-SY5Y human neuroblastoma cells and U-87 MG glioblastoma cells	5, 10, 15, 20 μg/mL	24 h	Inhibiting aberrant tau-like protein phosphorylation	GSK-3β, CDK5, JNK, MAPK, ERK1/MARK camkiiα, p-tau, APP, PS1, presenilin-2, apolipoprotein E4, and phosphatidylinositol binding clathrin assembly protein↓ TREM2 and IDE↑	Unknown	[121]
Xanthoceraside, husks of xanthoceras sorbifolia bunge	Male APP transgenic mice	0.02, 0.08, 0.32 mg/kg	6 months	Inhibiting aberrant tau-like protein phosphorylation	Tau protein at Ser396 and Ser404, pgsk-3β↓	Unknown	[59]
Theasaponin E1, green tea seeds	SH-SY5Y human neuroblastoma cells and U-87 MG glioblastoma cells	5, 10, 15, 20 μg/mL	24 h	Anti-inflammatory effect	IL-1β, IL-6, and TNF-α↓	NF-κB	[121]
Dioscin, dioscoreae nipponicae rhizomes	H_2_O_2_-induced SH-SY5Y cells Alcl_3_ combined with D-galactose-induced male C57BL/6 mice	In vitro: 2.5, 5 μM In vivo: 20, 40, 80 mg/kg	In vitro: 12 h In vivo: 4 weeks	Anti-inflammatory effect	RAGE, NOX4, IL-1β, IL-6, TNF-α, p-NF-κB(p-p65/NF-κB(p65), and AP-1↓	RAGE/NOX4	[144]
Platycodigenin, platycodon grandifloras	Aβ-induced BV2 microglia and primary cortical microglia	0.01–10 μM	4 days	Anti-inflammatory effect	Inos, TNF-α, Cox2, IL-1β phosphorylated p65, and p38↓ IL-10, IL-4, mannose receptor, arg 1, and chitinase-like proteins↑	PPARγ	[147]
Astragaloside IV, astmgali Radix,	LPS-induced BV2 microglial cells Male 5xFAD mice	In vitro: 10, 25, 50 μM In vivo: 10, 20 mg/kg	In vitro: 6 h In vivo: 3 months	Anti-inflammatory effect	IL-1β, COX-2, iNOS, and TNF-α↓	Unknown	[148]
Ginsenoside Rg5, ginseng	LPS-induced BV2 microglial cells	10, 30, 50 μM	1 h	Anti-inflammatory effect	Inos, IL-1β, COX-2, MMP-9 NO, TNF-α, NF-κB, and AP-1↓	MAPK and PI3K/Akt	[150]
Ginsenoside compound K, Panax notoginseng	LPS-induced BV2 microglial cells	0, 1, 2, 4, 6, 8, and 10 μM	2 h	Anti-inflammatory effect	TNF-α, IL-6, IL-1β↓ LRP1, NF-κB (p65)↑	NF-κB	[151]
P. Grandiflorum crude saponin, platycodon grandiflorum	Aβ-induced HT22 Hippocampal-derived neurons cells 5XFAD mouse	In vitro: 5, 10, and 20 μg/mL In vivo: 50 mg/kg	In vitro: 24 h In vivo: 3 weeks	Anti-inflammatory effect	P-NF-κB/NF-κB, COX-2↓ P-IκBα↑	NF-κB	[153]
Platycodin D, platycodon grandiflorum	Aβ-induced BV2 cells	0, 5, 10, 20, and 40 μM	24 h	Anti-inflammatory effect	TNF-α, IL-1β, and IL-6↓	TLR4/NF-κB	[155]
Notoginsenoside R2, Panax ginseng	Aβ-induced primary rat cortical neurons cells Male SAMP/8 mice Male SD rats	In vitro: 30 μM In vivo: 250 mg/kg	In vitro: Unknown In vivo: 8 weeks	Anti-inflammatory effect	COX-2↓	Mir-27a/SOX8/β-catenin	[159]
Saponins from bacopa floribunda, bacopa floribunda	Aβ-induced Eighty healthy BALB/c mice	50, 100, and 200 mg/kg	21 days	Anti-inflammatory effect	IL1β, TNF-α↓	Unknown	[161]
Astragaloside IV, astmgali Radix	Aβ-induced male ICR mice	20, 40, and 80 mg/kg	3 weeks	Anti-inflammatory effect	TNF-α, IL-1β, and IL-6↓	Unknown	[162]
Ginsenoside Rg1, ginseng	H_2_O_2_-inducedhippocampal neuron cells	1, 5, and 10 µM	24 h	Anti-inflammatory effect	NOX2, NLRP1↓	Unknown	[163]
Ginsenoside Rg1, ginseng	Male APP/PS1 mice	5, 10 mg/kg	12 weeks	Anti-inflammatory effect	NLRP1, IL-1β, and TNF-α↓	Unknown	[164]
Timosaponin BII, rhizoma anemarrhenae	LPS-induced PC12 cells LPS-induced C57BL/6J mice	In vitro: 0.313, 0.625, 1.25, 2.50, and 5.00 mg/mL In vivo: 20 mg/kg	In vitro: 12 h In vivo: 38 days	Anti-inflammatory effect	TNF-α, IL-1β↓	Unknown	[166]
Ginsenoside Rbl, ginseng	Aβ-induced male Wistar rats	10 mg/kg	4 weeks	Anti-inflammatory effect	COX-2, Ikb-α, Nnos↓	Unknown	[167]
Pseudoginsenoside-F11, ginseng	OA-induced male SD rats	2, 4, 8 mg/kg	4 weeks	Anti-inflammatory effect	TNF-α, IL-1β↓	Unknown	[168]
Cycloastragenol, astragalus radix	Aβ-induced male C57BL/6N mice	20 mg/kg/d	6 weeks	Anti-inflammatory effect	TNF-α and IL -1β↓	MAPK	[169]
Ginsenoside Rd, ginseng	Aβ-induced Sprague Dawley rat	10, 30 mg/kg	30 days	Anti-inflammatory effect	IL-1β, IL-6, TNF-α, and S100β↓ IL-10 and HSP70↑	Unknown	[170]
Hyoscyamoside, hyoscymus niger	Aβ-induced male Wistar rats	10 mg/kg	28 days	Anti-inflammatory effect	CDK11-P58, IL-6↓ IL-4↑	Unknown	[173]
Ginsenoside Rh2, ginseng	Scop-induced male ICR mice	12.5, 25 mg/kg	14 days	Antioxidative stress	MDA↓ GSH, SOD↑	Unknown	[195]
Saponins from bacopa floribunda, bacopa floribunda	Aβ-induced Eighty healthy BALB/c mice	50, 100, and 200 mg/kg	21 days	Antioxidative stress	ROS, MDA↓ GSH-Px, GSH, and CAT↑	Unknown	[161]
Ginsenoside Rd, ginseng	Aβ-induced Sprague Dawley rat	10, 30 mg/kg	30 days	Antioxidative stress	GSSG/GSH, HNE↓	Unknown	[170]
Ginsenoside Rg3, ginseng	D-gal-induced male wistar rats	20 mg/kg/d	60 days	Antioxidative stress	ROS↓ SOD, CAT, and GSH-Px↑	Unknown	[197]
Pseudoginsenoside-F11, ginseng	Aβ-induced male KM mice and APP/PS1 mice	0.32, 1.6, 8 mg/kg	4 weeks	Antioxidative stress	MDA↓ SOD, GSH-Px↑	Unknown	[198]
Ginsenoside Rg1, ginseng	Aβ-induced cortical neuron cells	2.5–10 μM	24 h	Antioxidative stress	ROS, H_2_O_2_↓ Cyt-c oxidase activity↑	Unknown	[199]
Huangjingsterol B, Stewed polygonatum cyrtonema hua.	Aβ-induced PC 12 cells	0, 1.25, 12.5, 25, 50, and 100 mm	2 h	Antioxidative stress	GSH-Px and SOD↑	Unknown	[200]
Solanum anguivi, solanum anguivi lam	Fe [2]^+^ and SNP-induced Rat brain synaptosome P2 fractions	10-200μg/mL	30 min	Antioxidative stress	ROS↓ Total thiol↑	Unknown	[201]
P. Grandiflorum crude saponin, platycodon grandiflorum	Aβ-induced HT22 hippocampal-derived neurons cells 5XFAD mouse	In vitro: 5, 10 and 20 μg/mL In vivo: 50 mg/kg	In vitro: 24 h In vivo: 3 weeks	Antioxidative stress	ROS↓ HO-1, SOD, CAT, and GSH-Px↑	Unknown	[153]
Notoginsenoside R1, panax notoginseng	Aβ-induced PC12 cells	1, 5, 10, 50, 100 μM	24 h	Antioxidative stress	ROS↓	Unknown	[202]
Protopanoxadiol saponins, leaves of p. Notoginseng	H_2_O_2_ or OGD-induced astrocytes and SH-SY5Ycells	2.5, 5, 10 μg/mL	24 h	Antioxidative stress	ROS Nrf2, HO-1, glutathione S-transferase pi 1↑	Unknown	[203]
Cycloastragenol, astragalus radix	Aβ-induced male C57BL/6N mice	20 mg/kg/d	6 weeks	Antioxidative stress	ROS, LPO↓ Nrf2, HO-1↑	Nrf2/HO-1	[169]
Ginsenoside Rg1, ginseng	H_2_O_2_-induced PC12 cells	0.1–10 μM	24 h	Antioxidative stress	Phosphorylation of NF-κB/p65 and IκB-α↓ IκB-α↑	NF-κB	[204]
Dioscin, dioscoreae nipponicae rhizomes	H_2_O_2_-induced SH-SY5Y cells Alcl_3_ combined with D-galactose-induced male C57BL/6 mice	In vitro: 2.5, 5 μM In vivo: 20, 40, 80 mg/kg	In vitro: 12 h In vivo: 4 weeks	Antioxidative stress	MDA, ROS↓ Nrf2, HO-1, SOD↑	RAGE/NOX4	[144]
Ginsenoside Rg1, ginseng	Aβ and D-gal-induced male tree shrews	7.5, 15, 30 mg/kg	8 weeks	Antioxidative stress	Nitrotyrosine, 8-OHG, and MDA↓ SOD, CAT, and GSH-Px↑	Wnt/GSK-3β/β-catenin	[205]
Esculentoside A, phytolaca esculenta	Triple transgenic AD mice	5, 10 mg/kg	8 weeks	Antioxidative stress	MDA, ROS↓ GSH, SOD↑	PPARγ	[207]
Ginsenoside Rb1, ginseng	Aβ-induced PC12 cells	0, 10, 20, 30, 40, and 50 μM	24 h	Antioxidative stress	MDA, ROS↓ cholesterol efflux↑	PPARγ	[208]
Platycodin D, platycodon grandiflorum	Aβ-induced BV2 cells	0, 5, 10, 20, and 40 μM	24 h	Antioxidative stress	ROS, MDA↓ SOD, NQO1↑	Nrf2/HO-1	[155]
Ginsenoside Re, ginseng	Aβ-induced SH-SY5Y cells	0, 5, 10, 20, 25, 30, 50, 75 μM	24 h	Antioxidative stress	ROS↓ NAD(P)H dehydrogenase 1, glutamate-cysteine, GSH, SOD, GSH-Px, GCLC↑	Nrf2/HO-1	[209]
Saikosaponin-D, Bupleurum falcatum	Glutamate-induced SH-SY5Y cells	0.5, 1, 5, 10 μM	6 h	Antioxidative stress	ROS, MDA↓ SO, gpx, CAT, Nrf2, and HO-1↑	PI3K	[212]
Astragaloside, Astragalus membranaceus	Aβ-induced primary cortical neuron Aβ-induced male Sprague Dawley rats	In vitro: 0, 50, 100, 200, 400 μg/mL In vivo: 40 mg/kg	In vitro: 24 h In vivo: 14 days	Antiapoptotic	Caspase-3↓	PI3K/AKT and MAPK (or ERK)	[227]
Notoginsenoside R2, Panax ginseng Male SAMP/8 mice Male SD rats	Aβ-induced primary rat cortical neurons cells	In vitro: 30 μM In vivo: 250 mg/kg	In vitro: Unknown In vivo: 8 weeks	Antiapoptotic	Caspase-3↓ SOX8, β-catenin↑	Mir-27a/SOX8/β-catenin	[159]
P. Grandiflorum crude saponin, Platycodon grandiflorum	Aβ-induced HT22 hippocampal-derived neurons cells 5XFAD mouse	In vitro: 5, 10 and 20 μg/mL In vivo: 50 mg/kg	In vitro: 24 h In vivo: 3 weeks	Antiapoptotic	Cyt-c release and caspase-9 and -3↓ Bcl-2 and Bcl-xl↑	MAPK	[153]
Astragaloside IV, Astmgali Radix	AβO-induced ht-22 cells AβO-induced Male C57BL/6 mice	In vitro: 0.1 μM In vivo: 10, 20, 40 mg/kg	In vitro: 24, 48 or 72 h In vivo: 1 week	Antiapoptotic	Caspase-3↓ PPARγ, BDNF↑	PPARγ/BDNF	[228]
Notoginsenoside R1, Panax notoginseng	Aβ-induced PC12 cells	1, 5, 10, 50, 100 μM	24 h	Antiapoptotic	Caspase-3, Bax, Bax/Bcl-2↓	MAPK	[202]
Ginsenoside Rg2, Ginseng	Aβ-induced SD rats	25, 50, 100 mg/kg	15 days	Antiapoptotic	Caspase-3↓ Bcl-2/Bax↑	PI3K/Akt	[229]
Ginsenoside Rg1, Ginseng	A double transgenic APP/PS1 rat model	AD rats were fed with 0.5% Rg1-enriched food	2 months	Antiapoptotic	Caspase-3, Grp78, p-JNK↓	JNK apoptotic pathway	[230]
Ginsenoside Rg1 and Acori graminei Rhizoma, Ginseng and Acori graminei Rhizoma	Aβ-induced primary hippocampal neurons Male SAMP8 mice	In vitro: 10 µmol/L In vivo: Ginsenoside Rg1 (7.5 mg/kg/day) and 0.1 g AGR/10 g of body weight	In vitro: 48 h In vivo: 3 weeks	Antiapoptotic	HO-1↓ Mir-873-5p↑	Unknown	[232]
Ginsenoside Rd, Ginseng	Aβ-induced Sprague Dawley rat	10, 30 mg/kg	30 days	Antiapoptotic	Caspase-3↓	Unknown	[170]
Ginsenoside Rg3, Ginseng	D-gal-induced male wistar rats	20 mg/kg/d	60 days	Antiapoptotic	Caspase-3, caspase-9, Bax, AIF, and Cyt C↓ Bcl-2↑	Unknown	[197]
Pseudoginsenoside-F11, Ginseng	Aβ-induced male KM mice and APP/PS1 mice	0.32, 1.6, 8 mg/kg	4 weeks	Antiapoptotic	JNK 2, p53, and cleaved caspase-3↓	Unknown	[198]
Cycloastragenol, Astragalus radix	Aβ-induced male C57BL/6N mice	20 mg/kg/d	6 weeks	Antiapoptotic	Bax, caspase-3, BIM↓ Bcl-2↑	Unknown	[169]
Lychee Seed Saponins, Lychee seed	Aβ-induced PC12 cells Aβ-induced male SD rats	In vitro: 0.95, 1.90, 3.80, 7.60 mg/L In vivo: 120, 240, 480 mg/kg	In vitro: 12 h In vivo: 28 days	Antiapoptotic	Bax, caspase-3↓ Bcl-2, Bcl-2/Bax↑	Unknown	[234]
Polygalasaponin F, Polygala japonica	Glutamate-induced primary culture of hippocampal neurons	2, 4, 6, 8, 10 μM	30 min	Antiapoptotic	Caspase-3↓	Unknown	[236]
